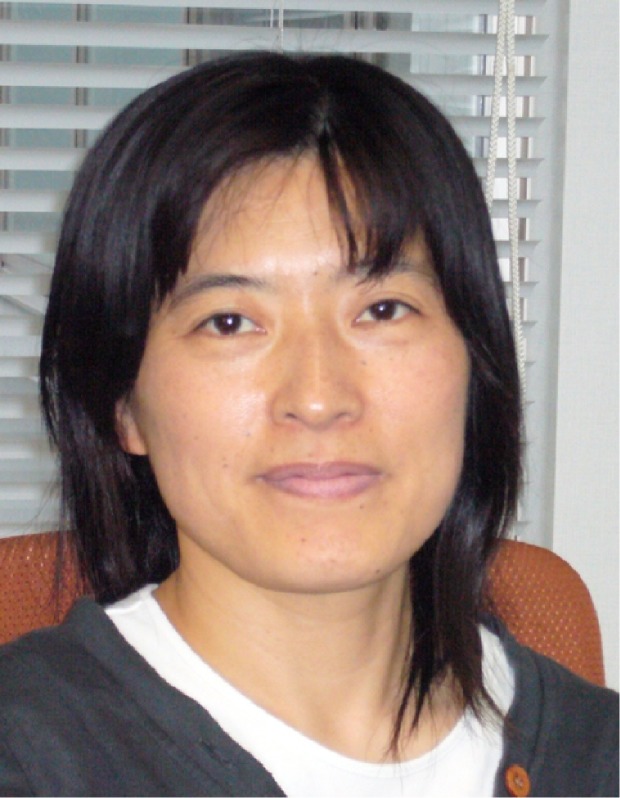# Message From the Editor-in-Chief

**DOI:** 10.2188/jea.JE20140245

**Published:** 2015-01-05

**Authors:** Manami Inoue

Dear Friends and Colleagues,

The official 2013 impact factor for the Journal of Epidemiology is 2.862. Our journal placed 29th among 162 journals in the category of Public, Environmental & Occupational Health, and top in the Asia-Pacific region. The proportion of self-citation was only 4%, indicating that ours is a sound and promising journal. The 10 most frequently cited papers in 2011 and 2012 are listed below.

I sincerely thank all editorial team members and reviewers for their outstanding effort in achieving this excellent result. I would particularly like to acknowledge the enormous contribution by our international associate editors from the Asia and Oceania regions, who first joined us in 2014.

Our editorial team looks forward to receiving new high-quality submissions from around the world in a broad range of topics in epidemiology. In this way, our journal can continue to advance research in basic and clinical science, public health science, and health policy.

Manami Inoue, MD, PhD Editor-in-Chief Journal of Epidemiology Project Professor AXA Department of Health and Human Security Graduate School of Medicine The University of Tokyo

**Figure:**